# An Investigation of the Best Methods of Destroying Lice and Other Body Vermin

**Published:** 1918-01

**Authors:** 


***An Investigation of the Best Methods of Destroying Lice and Other Body Vermin.*** By J. PARLANE KINLOCH, Lecturer, Univ. of Aberdeen. Abs. from *Brit. Med. Jour.*, June 3, 1916.

K. finds simple immersion of infested clothing in boiling water sufficient to kill lice. Of the various preparations for ridding clothing of lice, K. has tested volatile oils, phenol bodies, a mixture of ammoniated mercury, zinc oxide, and magnesium silicate, and also various commercial preparations including Dalmatian powder. Most of these were only slightly effective, and some, like Dalmatian powder, quickly lost their effectiveness when exposed to the air.

N. C. I. proved the most effective and lasting of all. K. also tested separately each of its ingredients (naphthaline 95 o/o, creosote 2 o/o, and iodoform 2 o/o). The tests showed that only the naphthaline and creosote were highly effective as insecticides. The iodoform, although only slightly so, was valuable for its adhesive qualities. K. believes, however, that the less costly magnesium silicate, which possesses the same adhesive qualities, but which is not an insecticide, might be substituded for iodoform without seriously impairing the effectiveness of the powder. There appeared to be no effective substitute for creosote or naphthaline. K. notes that commercial naphthaline is almost twice as effective as pure naphthaline, and attributes this fact to the presence of hydrocarbons and coal-tar derivatives.

The author has attempted to obviate the inconvenience resulting from the moisture in the N. C. I. powder, but finds that the process of drying weakens its effectiveness. It cannot be easily used in perforated tins, and he thinks it should be distributed in sealed tins because of the deteriorating influence of air.

